# Intestinal–Bone Axis Mediated by *Bifidobacterium*: Mechanistic Analysis and Therapeutic Potential in Osteoporosis

**DOI:** 10.3390/microorganisms14091985

**Published:** 2026-09-08

**Authors:** Fadong Li, Haoze Zhang, Boran Zhang, Xingwen Xie, Ning Li

**Affiliations:** 1 School of Clinical Chinese Medicine, Gansu University of Traditional Chinese Medicine, Lanzhou 730013, China; lifadong@163.com (F.L.); zhanghaoze@163.com (H.Z.);; 2 Affiliated Hospital of Gansu University of Traditional Chinese Medicine, Lanzhou 730020, China

**Keywords:** *Bifidobacterium*, osteoporosis, gut–bone axis, probiotics, gut microbiome, bone metabolism

## Abstract

Osteoporosis is one of the most prevalent metabolic bone diseases worldwide, and current pharmacological options are limited by adverse effects and are poorly suited to long-term use, underscoring the need for novel therapeutic targets. *Bifidobacterium*, one of the most representative beneficial bacterial genera in the human gut, has been linked to bone mineral density, and supplementation with specific strains improves bone metabolic parameters in animal models of osteoporosis. This narrative review examines the molecular mechanisms through which *Bifidobacterium* may protect bone via the gut–bone axis, encompassing four interconnected dimensions: reinforcement of the intestinal barrier, modulation of the immune network, remodeling of the gut microbiota, and production of bone-protective metabolites. The review makes three principal contributions. First, it establishes a four-tier mechanistic framework of *Bifidobacterium*-mediated regulation of osteoporosis in which the evidence is stratified into three categories—direct evidence from *Bifidobacterium*-specific studies, indirect evidence from other probiotics, and general mechanisms of gut microbiota-regulated bone metabolism—thereby strengthening the rigor of each argument and explicitly distinguishing evidence derived from *Bifidobacterium*-specific studies from that based on other probiotics or general gut-microbiota mechanisms throughout the review. Second, it systematically compares the osteoprotective efficacy of different *Bifidobacterium* strains (*Bifidobacterium longum*, *Bifidobacterium lactis*, *Bifidobacterium adolescentis*, etc.), highlighting the central importance of strain specificity. Third, it evaluates the strength of the evidence and identifies knowledge gaps for each mechanistic pathway. Most current evidence derives from cross-sectional studies and animal models; causal relationships await validation in large-scale prospective cohort studies and randomized controlled trials. In addition, functional disparities among strains and heterogeneity across clinical studies remain the core bottleneck in translating these fundamental findings into clinical practice.

## 1. Introduction

Osteoporosis is a metabolic bone disease characterized by reduced bone mass, deterioration of bone microarchitecture, and increased bone fragility, all of which substantially elevate the risk of fracture [1]. Epidemiological estimates indicate that more than 200 million people worldwide are affected, with a prevalence approximately three times higher in women than in men [2]. As the global population ages, the incidence of osteoporosis continues to rise, severely compromising patients’ quality of life and placing a substantial burden on health-care systems [2]. Because early symptoms are often atypical—chiefly mild pain and spinal deformity—early diagnosis is difficult, and the risk of fracture is markedly elevated [3]. Current pharmacotherapy relies mainly on bisphosphonates, selective estrogen receptor modulators, estrogen, and calcitonin, which act by promoting osteoblast function or suppressing osteoclast activity. However, bisphosphonates may impair renal function and induce or worsen hypocalcemia, whereas long-term use of selective estrogen receptor modulators and estrogen is associated with increased risks of thromboembolism and breast cancer. Safe, effective, and sustainable therapeutic strategies are therefore urgently needed for the prevention and treatment of osteoporosis.

The human body harbors a vast array of microorganisms [4], and the gastrointestinal tract is the most densely colonized site, accounting for roughly 70% of the total microbial load [5]. The combined genetic repertoire of the human gut ecosystem is approximately 150-fold larger than the human genome [6,7,8]. The gut microbiome is often regarded as a “hidden organ” whose structural and functional integrity supports essential physiological processes, including nutrient metabolism, immune regulation, and maintenance of the intestinal barrier [9,10]. The relationship between the gut microbiome and bone metabolism has attracted growing interest. The gut–bone axis is a bidirectional regulatory network in which the gut microbiome influences bone metabolic homeostasis through immune modulation, metabolite-mediated signaling, and regulation of the intestinal barrier, while changes in skeletal status in turn modulate the gut microecology. *Bifidobacterium* is the genus most commonly incorporated into probiotic formulations; it regulates bone metabolism through multiple pathways and, in preclinical models, has shown promise in inhibiting osteoclastogenesis and promoting bone formation [11]. This narrative review aims to elucidate the molecular mechanisms by which *Bifidobacterium* participates in the pathogenesis of osteoporosis through the gut–bone axis, to evaluate its potential value as a biomarker and therapeutic target, and to provide a theoretical foundation and translational directions for precision microbiome-based intervention in osteoporosis. Relative to earlier reviews of the gut–bone axis, the present article adds three distinctive elements: a four-tier mechanistic framework in which evidence is explicitly stratified by source; a systematic, strain-level comparison of osteoprotective efficacy; and an evidence-graded appraisal of each pathway, with the corresponding knowledge gaps explicitly identified. Given the early stage of this field, it should be emphasized that *Bifidobacterium*-based therapy for osteoporosis remains investigational; most supportive evidence derives from animal studies and cross-sectional observations, and large-scale randomized controlled trials with fracture-related endpoints are required to establish real-world efficacy and safety.

## 2. Gut–Bone Axis: Conceptual Framework and Regulatory Mechanisms

**Figure 1:**
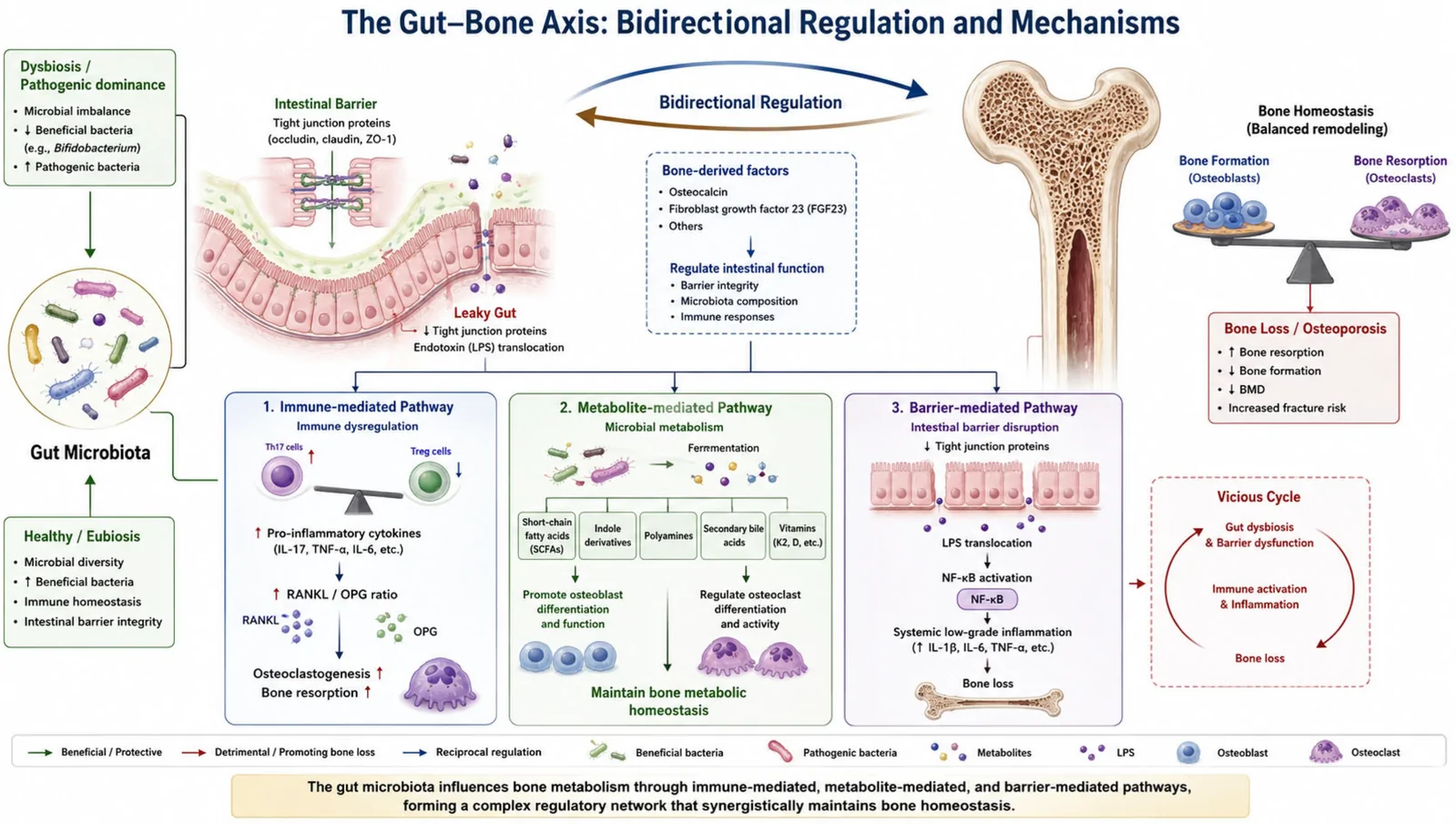
Bidirectional regulation and mechanistic pathways of the gut–bone axis. The gut–bone axis is a bidirectional regulatory network in which the gut microbiota and bone tissue mutually influence skeletal homeostasis. Gut dysbiosis disrupts intestinal barrier integrity, increases intestinal permeability, and facilitates lipopolysaccharide (LPS) translocation, activating NF-κB signaling and driving chronic low-grade inflammation, osteoclastogenesis, and bone loss. The gut microbiota regulates bone metabolism through three interconnected pathways: (i) the immune-mediated pathway, which modulates the Th17/Treg balance and RANKL expression; (ii) the metabolite-mediated pathway, in which microbe-derived compounds act via G protein-coupled receptor signaling, epigenetic regulation, and endocrine-like effects; and (iii) the barrier-mediated pathway, in which barrier disruption promotes endotoxin leakage and the release of inflammatory cytokines (e.g., TNF-α). For clarity, the schematic focuses on these three core pathways and their convergence on osteoclast and osteoblast activity.

Beyond its structural role, the skeleton serves as a reservoir for minerals and a site of hematopoiesis. Bone tissue undergoes continuous remodeling, with osteoblasts mediating formation and osteoclasts mediating resorption; the activity of both cell types is finely regulated by a range of cytokines [12]. When formation and resorption are in dynamic equilibrium, bone metabolism remains homeostatic; once this balance is disturbed, disorders of mineral metabolism and declining bone mineral density (BMD) ensue, ultimately culminating in metabolic bone diseases such as osteoporosis [13]. It was long assumed that bone metabolism is regulated chiefly by endocrine hormones (e.g., estrogen and parathyroid hormone), mechanical loading, and nutritional factors [14]. Yet the high bone mass observed under germ-free conditions does not represent healthy bone metabolism; it reflects immune hypoactivation and, consequently, insufficient osteoclastogenesis. When germ-free animals are recolonized with conventional microbiota, maturation of the immune system is accompanied by osteoclast activation and a subsequent decline in bone mass. The post-colonization changes in bone mass depend on microbial composition: a dominance of pathogenic bacteria accelerates bone loss, whereas a microbiota dominated by beneficial taxa such as *Bifidobacterium* preserves bone metabolic homeostasis through immune regulation [15,16]. These observations establish the gut microbiota as an important regulator of bone metabolism and give rise to the interdisciplinary field of “osteomicrobiology” [15]. The gut–bone axis emphasizes a bidirectional regulatory network linking the gut microbiome and the skeleton. Gut dysbiosis can promote bone loss by disturbing immune homeostasis, releasing pro-inflammatory mediators, and compromising the intestinal barrier. Conversely, patients with metabolic bone diseases such as osteoporosis often show impaired barrier function and altered microbiota composition, and a disrupted gut environment can further accelerate bone loss, creating a vicious cycle [17]. Bone-derived factors such as osteocalcin and fibroblast growth factor 23 can, in turn, regulate intestinal function through endocrine pathways [18,19]. Together, these observations delineate the bidirectional framework of the gut–bone axis and provide a rationale for microbiome-based strategies against osteoporosis. The gut microbiota influences bone metabolism through several interconnected routes, which can be grouped into three core dimensions: immune-mediated, metabolite-mediated, and barrier-mediated pathways. These three pathways do not operate independently; rather, they form a complex regulatory network that acts on bone metabolic homeostasis in a coordinated manner. Among them, the immune-mediated pathway is the core mechanism of gut–bone axis regulation. Skeletal and immune systems are closely intertwined, an interface known as “osteoimmunology” [20,21]. Gut microbiota imbalance can disrupt immune homeostasis, skewing CD4+ T-cell subsets toward pro-inflammatory T helper 17 (Th17) cells and away from regulatory T (Treg) cells, thereby increasing the secretion of pro-osteoclastogenic factors such as receptor activator of nuclear factor-κB ligand (RANKL) and promoting bone resorption [22]. The metabolite-mediated pathway involves bioactive compounds produced by microbial fermentation that directly regulate the differentiation and activation of osteoblasts and osteoclasts [23]. The barrier-mediated pathway centers on the integrity of the intestinal epithelial barrier: dysbiosis impairs tight-junction protein expression, producing a “leaky gut” that allows endotoxins such as lipopolysaccharide (LPS) to enter the bloodstream, where they activate nuclear factor-κB (NF-κB) signaling, induce systemic low-grade inflammation, and accelerate bone loss [24]. The conceptual framework of the gut–bone axis is illustrated in Figure 1.

## 3. Association Between Bifidobacterium and Osteoporosis

*Bifidobacterium* belongs to the phylum Actinobacteria [25]. It was first isolated by the French pediatrician Tissier from the feces of healthy breast-fed infants and is named for the characteristic bifurcated morphology of its cell termini [11]. The organism is a Gram-positive, obligately anaerobic, pleomorphic rod that uses a unique “bifid shunt” pathway, in which fructose-6-phosphate phosphoketolase metabolizes glucose to acetate and lactate in an approximately 3:2 ratio—a metabolic trait that confers a distinct ecological niche in the gut ecosystem [26]. More than 80 *Bifidobacterium* species have now been identified, of which roughly ten commonly colonize the human gut, including *Bifidobacterium longum* (*B. longum*), *Bifidobacterium breve* (*B. breve*), *Bifidobacterium lactis* (*B. lactis*), *Bifidobacterium adolescentis* (*B. adolescentis*), *Bifidobacterium bifidum* (*B. bifidum*), and *Bifidobacterium infantis* (*B. infantis*). These species differ markedly in genomic features, metabolic capacity, and host adaptability [27]. *Bifidobacterium* exerts diverse beneficial effects on host health, producing bioactive substances that include short-chain fatty acids (SCFAs), equol, exopolysaccharides (EPS), and B vitamins [28]. It also antagonizes pathogen colonization by competing for nutrients, producing antimicrobial substances, and modulating intestinal pH. These biological properties lay the foundation for its osteoprotective effects [29,30]. These functions are, however, markedly strain-specific: individual strains differ substantially in metabolite profiles and immunomodulatory capacity, and the properties of the genus as a whole should not be generalized (Table 1).

Several cross-sectional studies point to a potential correlation between *Bifidobacterium* abundance and BMD (Table 2). Gut microbial diversity and *Bifidobacterium* abundance were significantly lower in postmenopausal women with osteoporosis [31], and individuals with higher BMD showed significantly greater *Bifidobacterium* abundance than patients with osteoporosis [32]. Because such studies are correlational, they cannot establish causality, and age, diet [33], and environmental factors may substantially influence gut microbiota composition. In a mouse model of rheumatoid arthritis-induced osteoporosis, supplementation with *Bifidobacterium longum* FSHHK13M1 significantly improved BMD and trabecular bone microarchitecture and raised serum calcium levels [34]. *Bifidobacterium lactis* BL-99 significantly reduced pro-inflammatory cytokine levels (*p* < 0.05) and improved bone volume fraction and trabecular bone number in a dextran sulfate sodium-induced colitis model (*p* < 0.05) [29]. *Bifidobacterium longum* DSM 32947 increased whole-body and lumbar spine BMD in female mice [35]. Supplementation with *B. longum* raised serum osteocalcin levels, a marker of bone formation, and lowered C-terminal telopeptide of type I collagen, a marker of bone resorption [36]. Probiotic formulations containing *Bifidobacterium* improved bone turnover markers in postmenopausal women [31]. In aggregate, the available evidence suggests that reduced *Bifidobacterium* abundance is associated with the development and progression of osteoporosis; however, most of this evidence comes from cross-sectional studies and animal models, and large-scale prospective cohort studies and high-quality randomized controlled trials are needed to confirm causal relationships.

## 4. Mechanisms of Bifidobacterium in Regulating Osteoporosis

**Figure 2:**
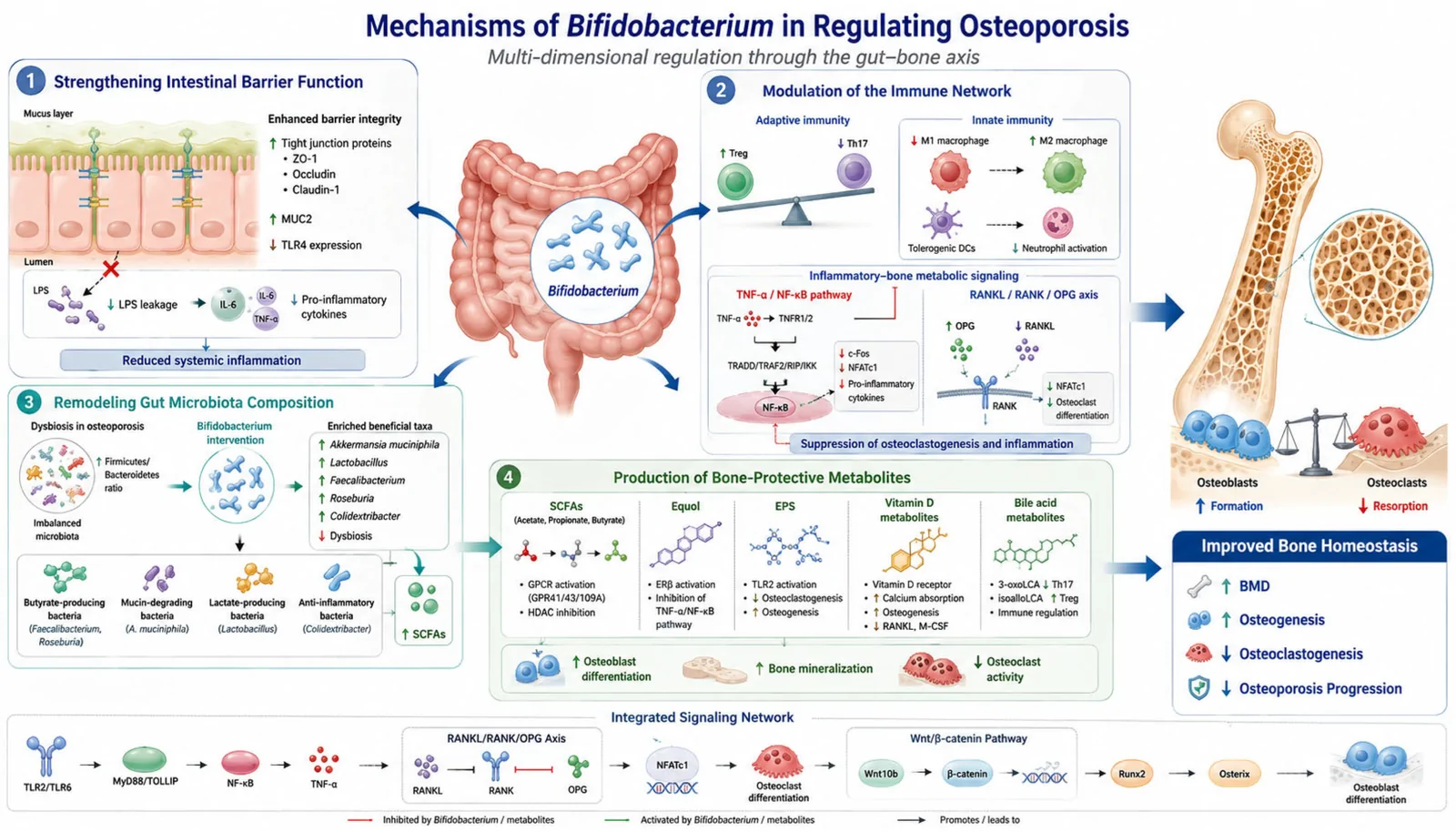
Integrated mechanisms of *Bifidobacterium* in regulating osteoporosis through the gut–bone axis. *Bifidobacterium* exerts osteoprotective effects through four synergistic dimensions. At the intestinal level, it strengthens the epithelial barrier by upregulating tight-junction proteins and mucin MUC2, thereby reducing LPS translocation and systemic inflammation. At the immune level, it promotes the Treg/Th17 balance and M2 macrophage polarization while suppressing M1-associated pro-inflammatory signaling, converging on inhibition of the TNF-α/NF-κB and RANKL/RANK/OPG axes. At the microbial ecosystem level, it enriches beneficial taxa such as *Akkermansia*, *Lactobacillus*, *Faecalibacterium*, and *Roseburia*. At the metabolite level, *Bifidobacterium*-derived or -modulated metabolites—including SCFAs, equol, exopolysaccharides, and vitamin D metabolites—regulate osteoblast differentiation and osteoclast activity via G protein-coupled receptor signaling (GPR41/43/109A), histone deacetylase inhibition, estrogen receptor activation, and osteogenic pathways (Wnt/β-catenin, Runx2/Osterix). Vitamin D receptor activation promotes calcium absorption and bone formation [37]. For clarity, only the principal effector cells and signaling nodes (Treg/Th17, M1/M2, TNF-α/NF-κB, RANKL/RANK/OPG) are depicted.

*Bifidobacterium* may protect bone by strengthening the intestinal barrier, regulating the immune network, remodeling gut microbiota composition, and producing bone-protective metabolites, with these actions forming a synergistic regulatory network. The sections below elaborate on the specific molecular mechanisms operating in each of the four dimensions. It bears emphasizing that the strength of the evidence varies across mechanisms and can be classified into three categories: experimental data obtained directly from *Bifidobacterium*-specific studies; findings from other probiotics (e.g., *Lactobacillus*), whose generalizability to *Bifidobacterium* requires verification; and general principles of gut microbiota-regulated bone metabolism, in which *Bifidobacterium* participates as one component rather than the sole executor. The integrated mechanisms are depicted in Figure 2.

### 4.1. Strengthening Intestinal Barrier Function

Disruption of intestinal barrier integrity is a critical contributor to systemic inflammation and bone loss [38]. The intestinal barrier comprises a mechanical component (tight-junction proteins), a chemical component (the mucus layer), and an immune component [10,39]. Intervention with *Bifidobacterium bifidum* FL228.1 and *Bifidobacterium animalis* F1-7 significantly upregulated the expression of the tight-junction proteins occludin, ZO-1, and claudin-2 [40]. Mechanistically, *B. bifidum* recruits TOLLIP through the Toll-like receptor (TLR)2/TLR6 receptor complex, activating transcription of the occludin gene [41,42]. *Bifidobacterium longum* K5 upregulated the messenger RNA expression of *ZO-1*, *occludin*, and *claudin-1* while downregulating *TLR4* expression [43]. At the chemical-barrier level, *B. bifidum* FL228.1 significantly increased colonic *MUC2* and *claudin-4* levels, raised the anti-inflammatory interleukin-10/pro-inflammatory interleukin-12 ratio, and enhanced mucus-layer integrity [44,45]. Once the barrier is breached, endotoxins such as LPS enter the bloodstream, triggering systemic inflammation and accelerating osteoclastogenesis [42]. *Bifidobacterium* preconditioning improved paracellular permeability in LPS-treated Caco-2 monolayers and reduced the production of interleukin-6 and tumor necrosis factor-α (TNF-α) [45,46]. *Bifidobacterium breve* CCFM1078 restored intestinal barrier integrity, reduced LPS translocation, and attenuated bone destruction in rats with collagen-induced arthritis [17]. Evidence for the barrier mechanism carries three limitations. First, with respect to model extrapolation, barrier assessments have relied mainly on in vitro Caco-2 monolayers and murine colitis models, which may not fully reproduce the gut dysbiosis characteristic of osteoporosis. Second, with respect to effect size, the 20–40% upregulation of tight-junction proteins may be insufficient to block osteoporosis-associated low-grade systemic inflammation, given that LPS circulates at picogram-per-milliliter levels. Third, with respect to regulatory mechanisms, whether *MUC2* regulation is strain-specific and whether long-term supplementation induces tolerance remain unresolved. Overall, the evidence for the barrier mechanism is of moderate strength, suggesting that it serves as an accessory rather than a dominant pathway in the osteoprotective effects of *Bifidobacterium*.

### 4.2. Modulation of the Immune Network

A central tenet of osteoimmunology is the intricate interplay between immune cells and bone cells. *Bifidobacterium* may exert anti-osteoporotic effects at both the adaptive (T-cell subset regulation) and innate (macrophage polarization, and neutrophil and dendritic cell modulation) levels, while simultaneously suppressing osteoclastogenesis through the TNF-α/NF-κB and RANKL/RANK/OPG signaling pathways.

#### 4.2.1. Adaptive Immunity Regulation

The balance among T-cell subsets is central to gut microbiota-regulated bone metabolism [14]. Th17 cells are pro-inflammatory T cells that secrete interleukin-17, TNF-α, and RANKL to promote osteoclast differentiation [47], whereas Treg cells exert immunosuppressive functions that restrain excessive bone resorption and maintain skeletal homeostasis [48]. *Bifidobacterium* strains such as *Bifidobacterium breve* AH1205 and *Bifidobacterium longum* AH1206 may protect bone by modulating gut microbiota composition, promoting Treg-cell differentiation, and suppressing Th17-cell expansion [49]. Part of the evidence linking Th17/Treg regulation to bone metabolism derives from probiotics other than *Bifidobacterium*. *Lactobacillus rhamnosus* GG protects skeletal health by maintaining the Th17/Treg balance and repairing estrogen deficiency-induced intestinal mucosal damage [50], and *L. rhamnosus* attenuates bone loss in ovariectomized (OVX) mice by modulating the Treg–Th17 balance [17]. These indirect findings are valuable references for probiotic bone protection through the T-cell axis, but whether they can be extrapolated to *Bifidobacterium* requires strain-specific experimental validation. The evidence chain for Th17/Treg regulation has important limitations. First, the key evidence derives largely from ovalbumin-induced Th17/Treg imbalance models, which are fundamentally distinct from the osteoporotic context; the regulatory weight of the Th17/Treg axis under osteoporotic conditions therefore remains to be established. Second, *Bifidobacterium* strains differ markedly in Treg-inducing capacity—the effect of *B. longum* AH1206 is significantly stronger than that of *B. breve* AH1205—so the immunomodulatory effects of one strain cannot be extrapolated to another. Third, the effect size of Th17/Treg regulation on bone metabolism varies considerably across OVX models, suggesting the existence of parallel dominant mechanisms such as TLR signaling or SCFA-mediated pathways.

#### 4.2.2. Innate Immunity Regulation

Innate immune cells also play an indispensable role in bone metabolism. Macrophages, as core components of innate immunity, exert dual effects on bone depending on their polarization state: classically activated (M1) macrophages promote RANKL-mediated osteoclast differentiation and bone resorption by secreting pro-inflammatory factors such as interleukin-1β, TNF-α, and interleukin-6, whereas alternatively activated (M2) macrophages inhibit osteoclastogenesis and promote osteoblast-mediated bone formation by secreting interleukin-10 and transforming growth factor-β [16,51,52]. Recent studies provide direct evidence that *Bifidobacterium*-mediated macrophage polarization influences bone metabolism. Zhang et al. demonstrated in an OVX mouse model that intervention with *B. bifidum* FL228.1 and *B. animalis* F1-7 significantly reduced the proportion of intestinal M1 macrophages, suppressed pro-inflammatory cytokine expression, and concurrently improved BMD and trabecular bone microarchitecture [53]. For the first time within a single experimental system, this finding provides preliminary evidence for the pathway “*Bifidobacterium* → M1 macrophage reduction → bone protection.” It should, however, be viewed as a plausible mechanistic link rather than an established causal chain, because macrophage depletion/rescue experiments, gene knockout models, and pathway-specific inhibitor studies are still needed to establish causality [54]. At the mechanistic level, Chen et al. demonstrated that *Bifidobacterium animalis* subsp. *lactis* LPL-RH suppressed osteoclastogenesis and bone loss in ovariectomized rats by downregulating the RANKL/TRAF6/NF-κB/NFATc1/TRAP signaling pathway [55]. Exopolysaccharides produced by *B. animalis* RH downregulate *RANK* expression, disrupt the *MITF*/*PU.1*/*NFATc1* transcriptional network, and simultaneously inhibit interleukin-6 and TNF-α production, exhibiting bidirectional activity that suppresses osteoclastogenesis and promotes osteogenesis [56].

Dendritic cells (DCs) bridge innate and adaptive immunity and are increasingly recognized as important regulators within the gut–bone axis. Gavzy et al. systematically reviewed the effects of *Bifidobacterium* on DCs: through its surface polysaccharides and metabolites, *Bifidobacterium* modulates the maturation status and cytokine-secretion profile of DCs and induces a tolerogenic phenotype, thereby promoting Treg-cell differentiation and suppressing Th17-cell responses [57]. This DC–Treg–Th17 regulatory axis may constitute an important immunological basis for the osteoprotective effects of *Bifidobacterium*; however, the direct role of DCs in *Bifidobacterium*-mediated bone regulation awaits validation in osteoporosis models. Neutrophils have drawn increasing attention for their bone-resorptive role in osteoporosis. Neutrophils can promote osteoclastogenesis directly by secreting TNF-α, interleukin-1β, and RANKL and can indirectly exacerbate bone resorption through the release of reactive oxygen species [58]. In animal models of osteoporosis, probiotic supplementation containing *Bifidobacterium* reduced intestinal neutrophil infiltration, improved intestinal barrier function, and attenuated bone loss [59], although direct evidence that *Bifidobacterium* specifically regulates neutrophil activity is still lacking. Natural killer (NK) cells have only recently come into focus in osteoimmune regulation. Kaur and Jewett [60] revealed that osteoclasts and probiotics containing *Bifidobacterium* synergistically activate NK cells: osteoclasts markedly expand NK cells and enhance their cytotoxic function, while probiotic components further promote NK-cell survival and cytokine secretion. *Bifidobacterium* has also been shown to increase NK-cell infiltration in tumors [57], suggesting a positive regulatory effect on NK-cell function. Nevertheless, direct evidence that *Bifidobacterium* regulates bone metabolism through NK cells is lacking, and the role of NK cells in the gut–bone axis represents a promising avenue for future research. Overall, the evidence for innate immune regulation varies considerably across cell types: macrophage polarization is supported by direct evidence validated in OVX models, dendritic cells are supported by reviews but lack osteoporosis-model validation, and neutrophils and NK cells rest on indirect evidence that requires osteoporosis-specific confirmation. The most robust finding to date is that *Bifidobacterium* is associated with reduced M1 macrophage polarization and inhibition of the NF-κB pathway in OVX models; even this association should be described as a plausible mechanistic link rather than an established causal chain, because macrophage depletion/rescue and knockout experiments have not yet been performed in the *Bifidobacterium*-osteoporosis context [54].

#### 4.2.3. Regulation of Inflammatory–Bone Metabolic Signaling Pathways

The TNF-α/NF-κB pathway is the core inflammatory cascade that drives osteoclastogenesis [61]. TNF-α, a potent pro-inflammatory cytokine produced by macrophages, activates a signaling cascade that includes TRADD, TRAF2, RIP, and IKK through binding to TNFR1 and TNFR2, ultimately activating the transcription factor NF-κB [62,63,64]. Activated NF-κB promotes c-Fos transcription and cooperates with NFATc1 to initiate the transcriptional program of osteoclast differentiation [65]. *Bifidobacterium* and its metabolites appear to attenuate activation of the TNF-α/NF-κB pathway, thereby reducing pro-inflammatory cytokine expression and preserving bone metabolic homeostasis. The RANKL/RANK/OPG axis is the core signaling system that governs osteoclastogenesis [66]. Within this axis, RANKL is produced by activated T and B cells and binds to the receptor activator of nuclear factor-κB (RANK) to trigger osteoclast differentiation, while osteoprotegerin (OPG) acts as a soluble decoy receptor. TNF-α, interleukin-6, and interleukin-1 can enhance osteoclast activity and suppress osteoblast function [34,62]. *B. longum* FSHHK13M1 significantly increased OPG expression and inhibited RANKL/RANK pathway activity, thereby suppressing osteoclast differentiation and activation [67]. *Bifidobacterium* can thus attenuate bone resorption by modulating the RANKL/RANK/OPG axis. These two pathways also exhibit regulatory crosstalk: under inflammatory conditions, TNF-α synergizes with RANKL to enhance osteoclastogenesis, whereas the ability of *Bifidobacterium* to target both pathways simultaneously confers a more comprehensive osteoprotective effect. Current evidence that *Bifidobacterium* inhibits the TNF-α/NF-κB pathway derives predominantly from in vitro experiments using RAW264.7 macrophages and LPS-induced acute inflammation models, which differ from the chronic low-grade inflammation characteristic of osteoporosis. Direct in vivo evidence in OVX models—ideally validated by gene knockout or pathway-specific inhibition—is still lacking for inhibition of the NF-κB pathway and for its synergistic weight with the RANKL pathway. This knowledge gap is one of the core bottlenecks in research on the osteoprotective mechanisms of *Bifidobacterium*.

### 4.3. Remodeling Gut Microbiota Composition

Gut microbiota dysbiosis is closely associated with the development of osteoporosis. An elevated Firmicutes/Bacteroidetes ratio is a potential biomarker of osteoporosis [30], and changes in the abundance of genera such as *Bacteroides*, *Dialister*, and *Eggerthella* are significantly associated with the disease [34]. *Bifidobacterium* intervention effectively remodels the gut microbiota: FL228.1 increased the abundance of *Lactobacillus* and *Colidextribacter*, and *B. longum* FSHHK13M1 increased the relative abundance of *Akkermansia muciniphila* and *Faecalibaculum rodentium* [68]. Functionally, the bacterial groups enriched after *Bifidobacterium* intervention fall into four types: (1) butyrate-producing bacteria (*Faecalibacterium*, *Roseburia*), whose butyrate regulates bone metabolism through histone deacetylase inhibition and G protein-coupled receptor activation [26,28]; (2) mucin-degrading bacteria (*A. muciniphila*), which promote mucus turnover [34]; (3) lactate-producing bacteria (*Lactobacillus*), which generate SCFAs synergistically; and (4) anti-inflammatory bacteria (*Colidextribacter*). Available evidence indicates that *Bifidobacterium* contributes a relatively modest share of the intestinal SCFAs pool, most of which is derived from other saccharolytic bacteria such as *Faecalibacterium* and *Roseburia*. Consequently, the osteoprotective effects attributed to SCFAs cannot be ascribed to *Bifidobacterium* alone, and the quantitative contribution of *Bifidobacterium* to SCFA-mediated bone regulation requires investigation using strain-specific tracing approaches [68]. Most of the observed microbiota changes after *Bifidobacterium* intervention, however, are based on 16S ribosomal RNA gene amplicon sequencing, which offers genus-level resolution only and cannot distinguish live from dead bacteria. The reported changes are predominantly correlational, and the causal direction remains unclear: the enriched genera, such as *Akkermansia* and *Faecalibacterium*, are themselves recognized beneficial bacteria, making it difficult to determine whether the osteoprotective effects result from the direct action of *Bifidobacterium* or from the indirect contribution of enriched taxa. The cross-feeding hypothesis currently rests on in vitro co-culture and metabolomic inference alone, with no in vivo isotope tracing experiments providing direct evidence. Upgrading 16S ribosomal RNA analysis to metagenomics, combined with metabolomics, is a necessary step for validating causal chains.

### 4.4. Production of Bone-Protective Metabolites

Beyond microbiota remodeling, the metabolites of *Bifidobacterium* play a central mediating role in gut–bone axis signaling and constitute an important molecular basis for its osteoprotective effects. SCFAs are the primary metabolites produced by *Bifidobacterium* fermentation of dietary fiber and consist mainly of acetate, propionate, and butyrate. SCFAs regulate bone metabolism through two pathways: the G protein-coupled receptor pathway, which activates GPR41, GPR43, and GPR109A to modulate Wnt/β-catenin and RANKL/RANK/OPG signaling [34,69,70], and the epigenetic pathway, in which butyrate inhibits histone deacetylases to promote osteogenic gene transcription [34,69,70]. SCFAs are not unique to *Bifidobacterium* but are shared metabolites of many gut bacteria. Butyrate also exerts epigenetic effects by inhibiting histone deacetylases, thereby promoting transcriptional activation of osteogenesis-related genes [68,71]. Propionate and butyrate induce metabolic reprogramming in osteoclasts, enhancing glycolysis while suppressing oxidative phosphorylation and thereby downregulating the expression of key osteoclast genes such as *TRAF6* and *NFATc1* [72]. Short-chain fatty acids can also inhibit LPS-induced expression of cytokines such as TNF-α and interferon-γ, increase interleukin-4 and interleukin-10 expression, induce colonic Treg-cell activation, reduce inflammatory cytokine production, and alleviate intestinal inflammation through interaction with GPR43. Butyrate additionally increases the expression of intracellular calcium transport proteins, promotes calcium absorption, limits parathyroid hormone production, and reduces bone resorption [73]. Propionate and butyrate can prevent bone loss in osteoporosis models by inhibiting osteoclastogenesis and modulating immunity [70].

The regulation of bone metabolism by SCFAs, however, is concentration-dependent and bidirectional. Physiological concentrations of SCFAs (in the millimolar range) promote osteoblast differentiation and mineralization, whereas sustained high concentrations (e.g., increased unprotonated forms at pH < 5.5) can promote bone resorption by activating osteoclast proton-sensing receptors [68]. This bidirectional effect implies that the net influence of short-chain fatty acids on bone metabolism depends heavily on local concentration and duration of exposure. Moreover, the SCFAs produced by *Bifidobacterium* account for only a fraction of the total intestinal pool, and the individual contribution of *Bifidobacterium* to bone metabolism currently lacks quantitative data from strain-specific ^13^C-labeled substrate tracing experiments. Equol is an intestinal metabolite of soy isoflavones, and *Bifidobacterium* is among the few microorganisms capable of producing it [74]. Equol possesses both estrogen receptor β agonistic and anti-inflammatory activities: on the one hand, it inhibits osteoclastogenesis by binding to estrogen receptor β and upregulating the OPG/RANKL ratio [75]; on the other, it inhibits the TNF-α/NF-κB pathway to reduce the pro-osteoclastogenic effects of inflammatory factors [76]. Equol treatment at 0.5 mg/day preserves bone mass, improves BMD, and increases bone mineral content in OVX mice [77]. Equol also exhibits potent estrogenic activity and may serve as a potential therapeutic agent for postmenopausal osteoporosis.

EPS are another important class of metabolites produced by *Bifidobacterium* [78]. EPS purified from *B. longum* (EPS-624) inhibits osteoclast differentiation from mouse bone marrow-derived macrophages while promoting osteogenic differentiation of human bone marrow-derived mesenchymal stem cells through activation of the TLR2 signaling pathway, exhibiting dual effects of promoting bone formation and inhibiting bone resorption [34]. *Bifidobacterium* can also influence vitamin D and bile acid metabolism and directly promote bone formation by activating multiple osteogenic signaling pathways. *B. longum* FSHHK13M1, confirmed through in vitro fecal fermentation models, significantly elevated vitamin D metabolite levels and increased serum 25(OH)D and 1,25(OH)_2_D concentrations in osteoporotic mice [79]. The active metabolite 1,25(OH)_2_D, by binding to the vitamin D receptor, activates calcium channel genes (e.g., *TRPV6*), calcium-binding protein genes, and osteogenesis-related genes (e.g., osteocalcin), promoting calcium absorption and bone formation while suppressing pro-osteoclastogenic factors such as RANKL and M-CSF [36,80]. Notably, *B. longum* FSHHK13M1 not only promotes bone formation through the vitamin D receptor pathway but also activates the *Wnt10b*/β-catenin and *Runx2*/*Osterix* osteogenic signaling pathways [79]. Activation of the *Wnt10b*/β-catenin pathway promotes the differentiation of mesenchymal stem cells toward osteoblasts while inhibiting adipogenic differentiation; *Runx2* and *Osterix*, as master transcription factors of osteogenic differentiation, coordinately initiate the transcriptional program of osteogenesis-related genes such as osteocalcin and type I collagen. Synergistic activation of these three pathways—vitamin D receptor, *Wnt10b*/β-catenin, and *Runx2*/*Osterix*—significantly enhances osteoblast differentiation and mineralization. Clinical studies further demonstrate that *B. longum* supplementation upregulates the expression of *SPARC* (osteonectin) and *BMP-2* (bone morphogenetic protein-2) [15]. Bile acid metabolites produced by the gut microbiota exert bidirectional effects on bone metabolism: 3-oxoLCA inhibits Th17 differentiation through RORγt binding, whereas isoalloLCA promotes Treg expansion by inducing mitochondrial reactive oxygen species [81]. The causal strength of the current evidence for *Bifidobacterium* metabolite-mediated bone protection varies: EPS [34] and vitamin D metabolism [34] have the most direct evidence base, validated by TLR2 knockout mice and vitamin D receptor pathway studies, respectively; evidence for equol derives primarily from indirect associations with soy isoflavone supplementation [74,75,76,77], and the actual contribution of *Bifidobacterium* to equol production in vivo has not been accurately quantified. SCFAs serve as core metabolic mediators, but *Bifidobacterium* contributes only a portion of the intestinal pool, most of which is derived from other saccharolytic bacteria. The precise contribution of *Bifidobacterium* to SCFA-mediated bone protection requires direct quantification by strain-specific metabolic tracing, and SCFAs should therefore be described as gut microbiota-derived metabolites with bone-regulatory properties rather than *Bifidobacterium*-specific mediators [68].

## 5. Bifidobacterium-Based Therapeutic Strategies for Osteoporosis

**Figure 3:**
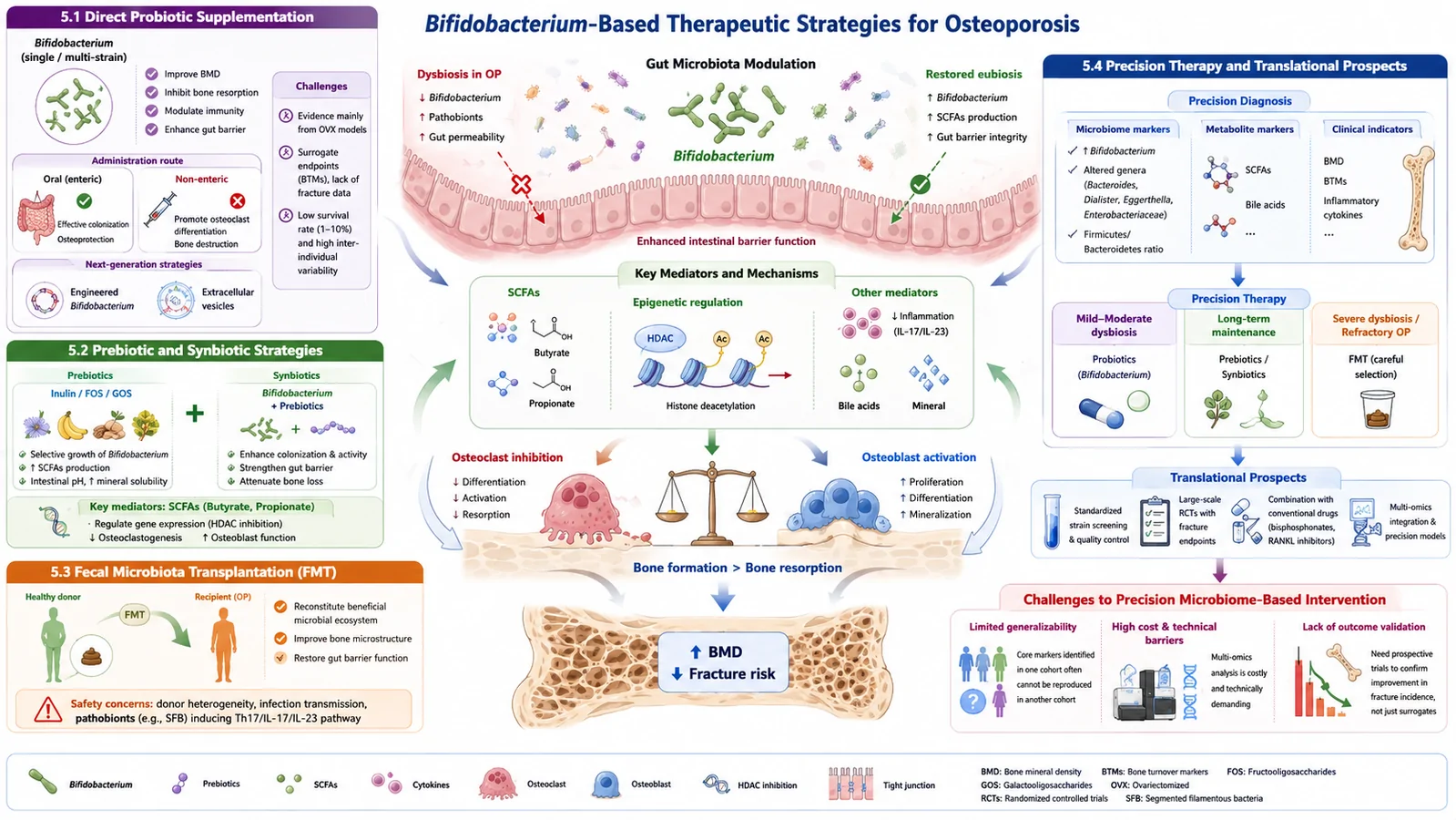
*Bifidobacterium*-based therapeutic strategies for the prevention and treatment of osteoporosis. *Bifidobacterium*-based interventions are a promising microbiome-targeted strategy that restores gut microbial homeostasis, enhances intestinal barrier integrity, and modulates immune–metabolic–bone signaling. Therapeutic strategies include direct probiotic supplementation, prebiotic and synbiotic interventions, fecal microbiota transplantation (FMT), and emerging precision microbiome-based therapies. Probiotic supplementation improves bone mineral density and inhibits osteoclast activity, although clinical translation is limited by strain-specific variability and colonization efficiency. Prebiotics and synbiotics selectively promote *Bifidobacterium* growth and SCFA-mediated regulation of bone cells, whereas FMT restores the global microbial ecosystem at the cost of donor heterogeneity and safety concerns. Microbiota-derived metabolites act through epigenetic regulation, G protein-coupled receptor signaling, and inflammatory pathway modulation to regulate osteoblast differentiation and osteoclastogenesis. Precision strategies integrate multi-omics profiling and clinical indicators (bone mineral density, bone turnover markers, inflammatory cytokines) to enable stratified intervention. For clarity, only the four main intervention categories and their shared downstream mediators are shown.

The regulatory relationship between the gut microbiome and bone metabolism has opened new avenues for the prevention and treatment of osteoporosis. Among the various microbiome-targeting strategies, probiotic therapy represented by *Bifidobacterium* shows potential for clinical translation because of its reported osteoprotective effects in preclinical studies, favorable safety profile, and suitability for long-term use. Most osteoprotective evidence, however, currently comes from preclinical studies, and the clinical efficacy and safety of *Bifidobacterium* preparations must be validated in large-scale randomized controlled trials before they can become an integral component of comprehensive osteoporosis management (Table 3). The therapeutic strategies discussed below are illustrated in Figure 3. It must be emphasized that the clinical application of *Bifidobacterium* in osteoporosis remains at an early stage: the available evidence is largely derived from animal models and small-scale or biomarker-based human studies, and rigorous efficacy and safety data from large-scale randomized controlled trials are still lacking.

### 5.1. Direct Probiotic Supplementation

Direct probiotic supplementation is the most straightforward microbiome-based intervention. Among probiotic intervention studies in osteoporosis, the genus *Bifidobacterium* is frequently investigated because of its repeatedly reported osteoprotective effects in animal studies. Systematic reviews indicate that *Bifidobacterium* accounts for 26% of the bacterial species used in animal experimental studies and shows potential efficacy in improving BMD and inhibiting bone resorption [28]. It should be noted, however, that this was a combined-intervention study with calcium and vitamin D co-supplementation and should therefore be interpreted as adjunctive or biomarker-level evidence rather than evidence for *Bifidobacterium* monotherapy. Clinical observations of a potential correlation between *Bifidobacterium* abundance and BMD support a role for this genus in osteoporosis pathophysiology [32]; nonetheless, heterogeneity across studies is high, and clinical evidence in established osteoporosis remains limited [32,82]. Multi-strain combination formulations exhibit synergistic therapeutic efficacy. VSL#3, which contains three *Bifidobacterium* species, four *Lactobacillus* species, and *Streptococcus thermophilus*, attenuated the decline in femoral BMD and trabecular thickness in ovariectomized animals, although these findings require validation in human clinical trials [32]. This multi-strain strategy leverages the core osteoprotective function of *Bifidobacterium* while optimizing the gut microenvironment with the assistance of auxiliary strains such as *Lactobacillus*. The route of administration significantly influences therapeutic efficacy: oral administration enables effective *Bifidobacterium* colonization of the gut and exerts osteoprotective effects, whereas non-enteric routes may induce bone destruction by promoting osteoclast differentiation [83].

Engineered probiotics and bacterial extracellular vesicles represent emerging strategies that remain at the preclinical or early-stage research phase. Studies on bacterial extracellular vesicles for osteoporosis treatment are still exploratory, with safety, efficacy, optimal dosing, and delivery protocols yet to be systematically evaluated [84]. Engineered *Bifidobacterium* platforms face substantial challenges, including biosafety validation, regulatory approval pathways, and production scalability, and must be clearly recognized as early-stage or preclinical approaches [83]. From a translational medicine perspective, advancing the osteoprotective effects of *Bifidobacterium* from animal models to clinical application faces multiple obstacles. First, the existing osteoprotective evidence is heavily concentrated in OVX mouse models, which simulate estrogen deficiency-induced bone loss but do not fully recapitulate the complex pathophysiology of senile osteoporosis or secondary osteoporosis. Second, existing clinical trials have used bone turnover markers as surrogate endpoints, with no randomized controlled trial reporting fracture incidence as a hard endpoint; positive findings from rodent models cannot be directly extrapolated to humans [81], and the quantitative association between bone turnover markers and fracture risk remains uncertain at the individual level. Third, only approximately 1–10% of orally administered viable *Bifidobacterium* survive gastric acid and bile exposure to reach the colon, producing substantial interindividual variability in effective dosage and rendering the dose–response relationship difficult to control precisely.

From a clinical perspective, several practical considerations must be addressed before *Bifidobacterium*-based interventions can be integrated into routine osteoporosis care. These include the selection of the most appropriate strain and formulation (single-strain vs. multi-strain), the optimal dose and treatment duration, the bioavailability and colonization efficiency of orally administered preparations, and the safety profile in older adults, who frequently present with polypharmacy and multiple comorbidities [81]. It is also important to determine whether *Bifidobacterium* should be used alone or as an adjunct to established interventions such as calcium and vitamin D supplementation, bisphosphonates, and weight-bearing exercise [37]. Well-designed head-to-head trials and pragmatic effectiveness studies are therefore needed to define the optimal implementation parameters and to ensure that probiotic therapy is positioned as a complementary—rather than alternative—component of comprehensive osteoporosis management.

### 5.2. Prebiotic and Synbiotic Strategies

Prebiotics are substrates selectively utilized by beneficial gut bacteria to confer health benefits [85]; they include mainly non-digestible carbohydrates such as inulin, fructooligosaccharides and galactooligosaccharides. Prebiotics exert a highly selective growth-promoting effect on *Bifidobacterium*, significantly enriching osteoprotective bacterial groups and indirectly enhancing *Bifidobacterium*-mediated osteoprotection by increasing SCFA production and lowering intestinal pH to promote mineral solubility [16]. This strategy—achieving therapeutic goals by feeding endogenous *Bifidobacterium*—offers the advantages of higher safety and better compliance in clinical application. The SCFAs produced by *Bifidobacterium* fermentation of dietary fiber are key mediators linking prebiotic intervention to skeletal health. Butyrate and propionate can regulate the expression of genes related to osteoclast differentiation by inhibiting histone deacetylase activity while stimulating osteoblast proliferation and mineralization [86]. Clinical studies have shown that SCFA supplementation improves bone turnover markers in postmenopausal women [87]. Synbiotics combine *Bifidobacterium* with prebiotics to form functional combinations that achieve synergistic effects through a live bacteria-plus-substrate model. The combined application of *Bifidobacterium* with fructooligosaccharides or galactooligosaccharides can more effectively enhance the intestinal colonization density and metabolic activity of *Bifidobacterium*, strengthen intestinal barrier function, and thereby attenuate bone loss [88]. Plant-based dietary patterns rich in dietary fiber offer a simple, feasible, and long-term sustainable nutritional intervention for patients with osteoporosis [89].

### 5.3. Fecal Microbiota Transplantation

Fecal microbiota transplantation (FMT) achieves holistic reconstitution of beneficial gut microbiota, including *Bifidobacterium*, by transplanting the complete gut microbial community from a healthy donor into the recipient [90]; it represents the highest level of microbiome-based intervention. The finding that fecal transplantation from osteoporosis patients accelerates bone loss in recipient animals experimentally suggests that gut microbiota dysbiosis may contribute to osteoporosis [91]. The osteoprotective effects of FMT have been preliminarily validated in aging animal models: transplantation of young donor microbiota significantly improved bone volume fraction, trabecular number, and trabecular thickness and restored intestinal barrier function in aged recipients [15]. Compared with single-strain preparations, FMT can reconstitute a more complete beneficial microbial ecological network in a single intervention, theoretically providing a more comprehensive regulatory effect [52]. Its clinical translation, however, requires careful evaluation of safety concerns. Certain components of the donor microbiota, such as segmented filamentous bacteria, may exacerbate bone destruction by inducing Th17-cell development through activation of the interleukin-17/interleukin-23 pathway; donor heterogeneity and the risk of infection transmission also require stringent control [92]. The choice of therapeutic strategy should therefore follow individualization principles: for patients with mild to moderate dysbiosis, probiotic preparations represented by *Bifidobacterium* can serve as a safe first-line option; prebiotic and synbiotic strategies suit patients requiring long-term maintenance therapy; and FMT may be reserved as a potent intervention for patients with severe dysbiosis-associated osteoporosis or those unresponsive to other treatments.

### 5.4. Precision Therapy and Translational Prospects

Characterization of gut microbiota profiles provides key tools for the precise implementation of *Bifidobacterium*-based therapy. Machine learning-based microbiome analyses have identified microbial markers (e.g., *Fusobacteria* and *Lactobacillaceae*) significantly associated with BMD [92], and *Bifidobacterium* abundance may serve as a core indicator for assessing osteoporosis risk and guiding treatment decisions [85]. At the genus level, changes in the abundance of *Bacteroides*, *Dialister*, *Eggerthella*, and members of the *Enterobacteriaceae* are also significantly associated with osteoporosis [85], and the Firmicutes/Bacteroidetes ratio can serve as an auxiliary diagnostic indicator [15]. Microbial metabolites also carry therapeutic guidance value: serum total bile acids show a potential correlation with BMD and a negative correlation with bone resorption markers [93]. A precision diagnostic and therapeutic model integrating multi-omics data, including microbiomics and metabolomics, will drive the transition of probiotic therapy from empirical use to individualized precision intervention [82]. Probiotic therapy for osteoporosis, represented by *Bifidobacterium*, is currently in a critical transitional phase from proof-of-concept to efficacy confirmation. Existing randomized controlled trials have demonstrated heterogeneous effects of probiotic supplementation on bone metabolic indicators in postmenopausal women, primarily attributable to differences in strain selection, dosage, treatment duration, and individual microbiota composition [94,95]. Standardized clinical trials are accelerating the accumulation of evidence: a double-blind, randomized, placebo-controlled trial in early postmenopausal women has adopted dual-energy X-ray absorptiometry to evaluate the efficacy of *Bifidobacterium* preparations on BMD [96]. Future research should focus on the following directions: conducting multicenter, long-term follow-up clinical trials to establish level I evidence; establishing standardized systems for strain screening and quality control; exploring synergistic regimens that combine *Bifidobacterium* with conventional drugs such as bisphosphonates and RANKL inhibitors; and constructing precision diagnostic and therapeutic decision models based on microbiota characteristics and metabolic phenotypes [37]. Precision microbiome-based intervention, however, currently faces three major challenges: the limited cross-cohort generalizability of existing microbial classification models, in which core microbial markers identified in one cohort often cannot be reproduced in another; the high cost and technical threshold of integrated multi-omics analysis, which limits clinical implementation; and the need for prospective clinical trials to determine whether treatment decisions based on microbiota characteristics improve clinical outcomes (fracture incidence) rather than merely surrogate endpoints (BMD or bone turnover markers). In this context, the trabecular bone score—a gray-level textural index of bone microarchitecture derived from lumbar spine dual-energy X-ray absorptiometry images—has emerged as a complementary measure of bone quality that provides information not captured by areal BMD [97,98]. TBS has been shown to predict fragility fractures independently of BMD and FRAX probability in large prospective cohorts [97,98]. We therefore propose that TBS be incorporated as a supplementary outcome in future clinical studies of microbiome-based interventions for osteoporosis, which could strengthen the translational relevance of this field.

## 6. Conclusions

The bidirectional regulatory network between the gut microbiome and the skeletal system has provided a novel perspective for understanding the pathophysiology of osteoporosis. This review has articulated the scientific concept of the gut–bone axis, dissected the molecular mechanisms by which *Bifidobacterium* regulates osteoporosis, and explored the clinical translational prospects of microbiome-targeted therapeutic strategies centered on *Bifidobacterium*. *Bifidobacterium* may protect bone through four synergistic dimensions: strengthening intestinal barrier function, regulating the immune network, remodeling gut microbiota composition, and producing bone-protective metabolites. At the barrier level, it may upregulate tight-junction protein expression, enhance mucus-layer protection, and reduce endotoxin entry into the bloodstream; at the immune level, it may modulate the Th17/Treg balance, inhibit the TNF-α/NF-κB and RANKL/RANK/OPG pathways, and regulate macrophage polarization; at the microbiota level, it may increase beneficial bacterial abundance and suppress pathogenic colonization; and at the metabolite level, SCFAs, equol, and EPS produced by *Bifidobacterium* may regulate bone metabolism. Probiotic therapy centered on *Bifidobacterium*, combined with prebiotic support and FMT, may offer new avenues for the prevention and adjunctive management of osteoporosis, although further clinical validation is required. It is important to underscore that *Bifidobacterium*-based therapy for osteoporosis is still in its infancy, and its routine clinical use should await confirmation of efficacy and long-term safety in large-scale randomized controlled trials.

Significant knowledge gaps and technical bottlenecks, however, remain between fundamental discoveries and clinical applications. First, most current evidence comes from correlational studies and animal models, and the causal relationship between reduced *Bifidobacterium* abundance and osteoporosis requires rigorous validation in human studies. Second, current research often employs the generalized concept of the *Bifidobacterium* genus while neglecting functional differences among species and even strains; the osteoprotective efficacy and targets of different species, such as *B. longum*, *B. lactis*, and *B. breve*, may differ substantially. Third, existing randomized controlled trials show significant heterogeneity in strain selection, dosage, treatment duration, and efficacy endpoints, severely constraining the integration of evidence-based data and the formulation of clinical guidelines. Future research should focus on the following directions: establishing high-throughput screening platforms for the osteoprotective activity of *Bifidobacterium*, combined with comparative genomics to identify functional gene modules and provide molecular markers for superior strain selection; developing personalized strain-matching strategies based on host microbiota background; establishing quality-control standards for preparations and determining optimal dosage ranges and effective treatment courses; and conducting multicenter clinical trials with changes in BMD and fracture incidence as core endpoints. The integration of multi-omics and machine learning may further enable efficacy prediction models based on baseline microbiota characteristics and metabolic phenotypes, driving the transition of probiotic therapy from empirical use to individualized intervention. As these key issues are progressively resolved, microbiome-based intervention strategies centered on *Bifidobacterium* may provide complementary approaches for comprehensive osteoporosis management, pending rigorous clinical validation. Clinicians and patients should therefore regard *Bifidobacterium*-based strategies as an adjunctive and experimental approach at the present time, with decisions on strain selection, dosage, treatment duration, and combination with calcium, vitamin D, or exercise individualized on the basis of the limited available evidence.

## 7. Search Strategy and Study Selection

This narrative review was conducted in line with the principles of the Scale for the Assessment of Narrative Review Articles (SANRA). We systematically searched PubMed, Web of Science, Scopus, and Embase from inception to June 2026 using combinations of the following search terms: “*Bifidobacterium*”, “probiotics”, “gut microbiota”, “osteoporosis”, “bone mineral density”, “bone metabolism”, and “gut–bone axis.” Original research articles, systematic reviews, and meta-analyses published in English or Chinese that examined the relationship between *Bifidobacterium*/probiotics and bone metabolism were considered. Conference abstracts, dissertations, non-peer-reviewed preprints, and studies without bone-related outcomes were excluded. Two authors (F.L. and H.Z.) independently screened titles and abstracts and subsequently assessed full texts, and disagreements were resolved by discussion with a third author (X.X.). The retrieved evidence was stratified into three tiers according to its source—direct evidence from *Bifidobacterium*-specific studies, indirect evidence from other probiotics, and general mechanisms of gut-microbiota-regulated bone metabolism—and the strength of each mechanistic pathway was graded accordingly.

## Figures and Tables

**Table 1:** Experimental and clinical evidence for the osteoprotective effects of different *Bifidobacterium* strains.

Strain	Route	Dose (CFU/d)	Duration	Model	Key Findings
*B. longum* FSHHK13M1	Oral (gavage)	1 × 10^9^	3 wk	RA-induced OP mice	BMD ↑, trabecular microarchitecture ↑, serum Ca ↑; OPG ↑, RANKL ↓, Wnt10b ↑ [Single strain; no co-administered Ca/VitD reported; endpoints: BMD, trabecular parameters, BTMs]
*B. lactis* BL-99	Oral (gavage)	1 × 10^9^	4 wk	DSS colitis-OP mice	BV/TV ↑, trabecular bone ↑; TNF-α ↓, IL-1β ↓, IL-6 ↓ [Single strain; DSS model; no Ca/VitD co-administration; endpoints: bone microarchitecture, inflammatory cytokines]
*B. longum* DSM32947	Oral (gavage)	1 × 10^9^	8 wk	OVX female + sham mice	Whole-body BMD ↑, BMC ↑, lumbar BMD ↑ [Single strain; no co-administered Ca/VitD; endpoints: DXA-based BMD and BMC]
*B. adolescentis* CCFM1447	Oral (gavage)	1 × 10^9^	6 wk	RA-induced OP mice	Trabecular number ↑, BMD ↑; 1,25(OH)_2_D ↑ [Single strain; no Ca/VitD co-administration; endpoints: BMD, trabecular parameters, vitamin D metabolites]
*B. bifidum* FL228.1	Oral (gavage)	1 × 10^9^	8 wk	OVX mice	Occludin ↑, ZO-1 ↑, MUC2 ↑, M1 macrophages ↓ [Single strain; OVX model without Ca/VitD; endpoints: tight junction proteins, macrophage polarization]
*B. lactis* Probio-M8^+^	Oral	2 × 10^10^	6–12 mo	Postmenopausal OP patients	VD3 ↑, PTH ↓ [Single strain; co-administered calcium and calcitriol; adjunctive/biomarker-level evidence; endpoints: BTMs; no BMD or fracture data]

Abbreviations: ↑, increase; ↓, decrease; BMD, bone mineral density; BMC, bone mineral content; BV/TV, bone volume/tissue volume; OPG, osteoprotegerin; RANKL, receptor activator of nuclear factor-κB ligand; RA, rheumatoid arthritis; OVX, ovariectomized; DSS, dextran sulfate sodium; TNF-α, tumor necrosis factor-α; IL, interleukin; VD3, vitamin D3; PTH, parathyroid hormone; ZO-1, zonula occludens-1; MUC2, mucin 2. Unless otherwise specified, strains were administered alone without co-administration of calcium or vitamin D. The Probio-M8 study represents an adjunctive clinical intervention with calcium and calcitriol co-supplementation.

**Table 2:** Summary of evidence for the association between *Bifidobacterium* and osteoporosis.

Evidence Type	Study Population/Model	Key Findings
Cross-sectional	Postmenopausal OP patients	Bifidobacterium abundance ↓ vs. controls; diversity positively correlated with BMD
Cross-sectional	High BMD vs. OP women	Bifidobacterium abundance higher in high BMD group; correlated with lumbar BMD (r > 0.3)
Animal experiment	RA-OP mice	*B. longum* FSHHK13M1: BMD ↑, trabecular bone ↑, Ca ↑
Animal experiment	DSS colitis-OP mice	*B. lactis* BL-99: BV/TV ↑, TNF-α ↓, IL-6 ↓ (*p* < 0.05)
Animal experiment	OVX-OP mice	*B. longum* DSM32947: whole-body BMD ↑, BMC ↑, lumbar BMD ↑
Animal experiment	RA-OP mice	*B. adolescentis* CCFM1447: trabecular bone ↑, VD metabolism enriched
Animal experiment	OVX mice	*B. bifidum* FL228.1: Occludin ↑, ZO-1 ↑, MUC2 ↑, M1 ↓
Clinical intervention (adjunctive)	Postmenopausal OP patients	*B. lactis* Probio-M8: OCN ↑, CTX-I ↓, VD3 ↑
Meta-analysis	Postmenopausal women	Probiotics containing Bifidobacterium → CTX-I ↓ (SMD = −1.51, 95%CI: −1.88 to −0.41)

Abbreviations: ↑, increase; ↓, decrease; RA, rheumatoid arthritis; DSS, dextran sulfate sodium; OVX, ovariectomized; BMD, bone mineral density; BV/TV, bone volume/tissue volume; BMC, bone mineral content; VD, vitamin D; OCN, osteocalcin; CTX-I, C-terminal telopeptide of type I collagen; SMD, standardized mean difference; CI, confidence interval.

**Table 3:** Comparison of microbiome-targeted therapeutic strategies for osteoporosis based on *Bifidobacterium*.

Strategy	Regimen	Mechanism	Key Advantages	Limitations
Probiotic supplementation	Single or multi-strain (oral, 10^9^–10^11^ CFU/d, 4 wk–12 mo)	Intestinal barrier ↑; Th17/Treg balance; SCFAs/Equol → RANKL pathway	Good safety; consistent effects; suitable for long-term use	Functional differences among strains (3–5 fold); 1–10% reach colon
Prebiotics and synbiotics	FOS/GOS/inulin (8–15 g/d) or Bifidobacterium + FOS/GOS	Selective Bifidobacterium enrichment (5–10 fold); SCFAs → pH ↓→Ca absorption ↑	Highest safety; good compliance; no viable delivery needed	Interindividual variability; limited evidence for prebiotics alone on BMD
FMT	Healthy donor microbiota → oral capsule or colonoscopy	Complete microecological reconstitution; ZO-1/Occludin ↑; Th17 ↓	Most comprehensive remodeling; theoretically full axis restoration	Donor heterogeneity; infection risk; no long-term safety data
Engineered bacteria and BEVs	Engineered Bifidobacterium (BMP-2) or BEV delivery	BEVs: TLR2 ligands → osteoclast ↓; engineered bacteria: precision targeting	Precision regulation; BEVs avoid colonization risk; early-stage proof-of-concept in animal models	Biosafety unvalidated; unclear regulatory path; high cost; still in preclinical/early-stage research
Precision intervention	ML + multi-omics → personalized strain matching	Baseline microbiota predicts efficacy; F/B ratio and Bifidobacterium as biomarkers	Improved consistency; avoids ineffective interventions	Cross-cohort generalizability issues; high multi-omics cost

Abbreviations: ↑, increase; ↓, decrease; CFU, colony-forming units; FOS, fructooligosaccharides; GOS, galactooligosaccharides; FMT, fecal microbiota transplantation; BEV, bacterial extracellular vesicle; BMP-2, bone morphogenetic protein-2; TLR2, Toll-like receptor 2; SCFA, short-chain fatty acid; Th17, T helper 17 cell; ZO-1, zonula occludens-1; F/B, Firmicutes/Bacteroidetes; ML, machine learning.

## Data Availability

No new data were created or analyzed in this study. Data sharing is not applicable to this article.

## Abbreviations

BMD	bone mineral density
SCFA	short-chain fatty acid
Th17	T helper 17 cell
Treg	regulatory T cell
TNF-α	tumor necrosis factor-α
NF-κB	nuclear factor-κB
RANKL	receptor activator of nuclear factor-κB ligand
RANK	receptor activator of nuclear factor-κB
OPG	osteoprotegerin
LPS	lipopolysaccharide
OVX	ovariectomized
NK	natural killer
M1	classically activated macrophage
M2	alternatively activated macrophage
DC	dendritic cell
TLR	Toll-like receptor
EPS	exopolysaccharides
FMT	fecal microbiota transplantation
SANRA	Scale for the Assessment of Narrative Review Articles
